# A survey on the perception of allergy specialists about the reimbursed grass pollen tablets for seasonal allergic rhinitis in Italy

**DOI:** 10.1186/s12948-017-0071-6

**Published:** 2017-08-15

**Authors:** Ilaria Massaro, Oliviero Rossi, Cristoforo Incorvaia, Carlo Lombardi

**Affiliations:** 1 Cell Therapy and Transfusion Medicine, Azienda Ospedaliero Universitaria Careggi, Florence, Largo Brambilla 1, 50134 Florence, Italy; 2 Allergy Unit, Azienda Ospedaliero Universitaria Careggi, Florence, Florence, Italy; 3 Cardiac/Pulmonary Rehabilitation, Centro Specialistico Gaetano Pini/CTO, Milan, Italy; 4 Allergy and Pneumology Departmental Unit, Fondazione Poliambulanza Hospital, Brescia, Italy

## Abstract

**Background:**

Sublingual immunotherapy (SLIT) is a feasible option to classical subcutaneous immunotherapy to treat respiratory allergy and is increasingly prescribed in Europe. However, the lack of reimbursement may limit its prescription. In 2015, the 5-grass pollen tablets was authorized by the European Medicine Agency to treat grass-pollen induced rhinitis and was approved in Italy for full reimbursement. We evaluated the opinions of allergy specialists after the availability of the reimbursed 5-grass pollen tablets.

**Methods:**

A multiple choice questionnaire composed by six questions was used to assess the specialists opinion. The questionnaire was uploaded on the free access online platform SurveyMonkey. The link to access the platform was sent to all members of the Società Italiana di Asma, Allergologia e Immunologia Clinica (SIAAIC). The access to the questionnaire was anonymous. At survey ending, the access was closed and data were downloaded directly from SurveyMonkey website.

**Results:**

The questionnaire was filled by 70 allergists. The majority of allergists felt as most important the concept of SLIT as a drug, the content of allergen extract mirroring the natural exposure, the pre-coseasonal schedule as the most patient’s oriented, the very good profile of tolerability and safety, the importance of the build-up phase, and the importance of checking the patient after starting immunotherapy.

**Conclusions:**

The opinions of the Italian allergy specialists about the reimbursed 5-grass-pollen tablets are very positive and make likely an appropriate prescription of SLIT for grass-pollen induced rhinitis in the next years.

## Background

Allergen immunotherapy (AIT) is an efficacious, evidence-based treatment for allergic rhinitis [1]. Sublingual immunotherapy (SLIT) is a feasible option to classical subcutaneous immunotherapy (SCIT), that has its main advantages over SCIT in a better safety and in a lower cost [2, 3]. Actually, SLIT is increasingly prescribed also in countries where SCIT has been dominant, such as Germany [4] and is the most used form of AIT in Italy [5]. The allowance of allergen extracts for AIT by the different regional health systems in Italy is uneven, and in a study that evaluated the factors influencing the AIT prescription by a questionnaire submitted to about 450 specialists, the cost of treatment and the reimbursement were listed among the factors possibly influencing the prescription [6]. In 2015, the 5-grass pollen tablets, based on the fulfillment of all the requirements by the European Medicine Agency (EMA), that resulted in the authorization of the product to treat grass-pollen induced rhinitis, was approved by the Agenzia Italiana per il Farmaco (AIFA) for full reimbursement, i.e. class A, as for drugs [7]. 

We aimed this study at evaluating the opinions of allergy specialists after the availability of the reimbursed 5-grass pollen tablets.

## Methods

A multiple choice questionnaire developed by a group of allergists of the Società Italiana di Asma, Allergologia e Immunologia Clinica (SIAAIC) and composed by six questions (reported in Table 1) was used to assess the specialists opinion. The questionnaire was uploaded on the free access online platform SurveyMonkey (http://www.surveymonkey.com) on March 1 2016. The link to access the platform and the optional answers to the questionnaire was sent to all SIAAIC members by the SIAAIC scientific secretary. The access to the questionnaire was anonymous. On April 7 2016 the access was closed and data were downloaded directly from SurveyMonkey website.

**Table 1:** Questions and answers choices

Questions	Choices
According to your experience, what was changed by the reimbursement of the grass-pollen tablets for seasonal rhinitis?	a. Accessibility to sublingual immunotherapy b. Possibility to adequately treat patients with moderate to severe seasonal rhinitis c. Possibility to treat all ARIA stages of rhinitis d. The concept of AIT as a drug
According to your opinion, how important is that the content of the allergen extract mirror the patient’s natural exposure?	a. Very important b. Not important c. I do not know
Which is the treatment schedule for grass pollen-induced rhinitis more oriented toward patient’s preference?	a. Perennial b. Pre-coseasonal c. Indifferent
According to your experience, the registered sublingual immunotherapy has a tolerability and safety profile	a. Very good b. Good c. Quite low
In performing sublingual immunotherapy, is the build-up phase important?	a. Yes b. No c. I do not know
After starting registered sublingual immunotherapy, do you check the patient after some weeks/months?	a. Always b. Sometimes c. Never

## Results

**Fig. 1:**
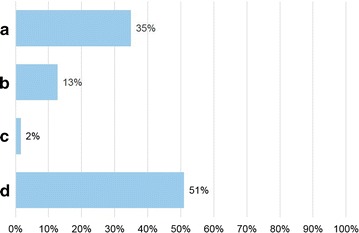
Answers to the question “According to your experience, what was changed by the reimbursement of the grass-pollen tablets for seasonal rhinitis?”

Seventy allergists anonymously filled in the questionnaire. Figures 1 2, 3, 4, 5 and 6 depict the data obtained. The majority of allergists chose as most important the concept of AIT as a drug for question 1, the content of allergen extract mirroring the natural exposure for question 2, the pre-coseasonal schedule as the most patient’s oriented for question 3, a very good profile of tolerability and safety for question 4, the importance of the build-up phase for question 5, and the importance of checking the patient after starting immunotherapy for question 6.

**Fig. 2:**
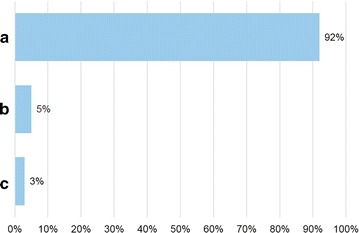
Answers to the question “According to your opinion, how important is that the content of the allergen extract mirror the patient’s natural exposure?”

**Fig. 3:**
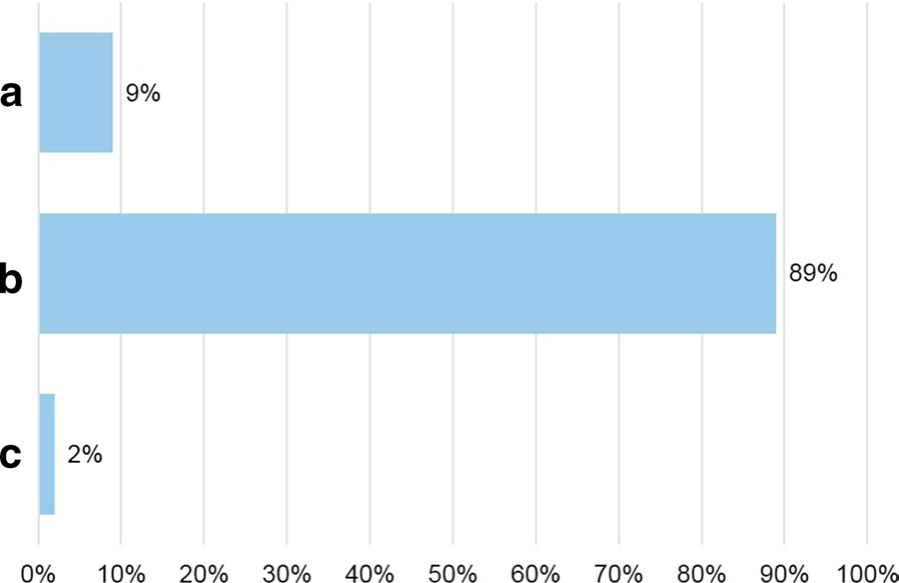
Answers to the question “Which is the treatment schedule for grass pollen-induced rhinitis more oriented toward patient’s preference?”

**Fig. 4:**
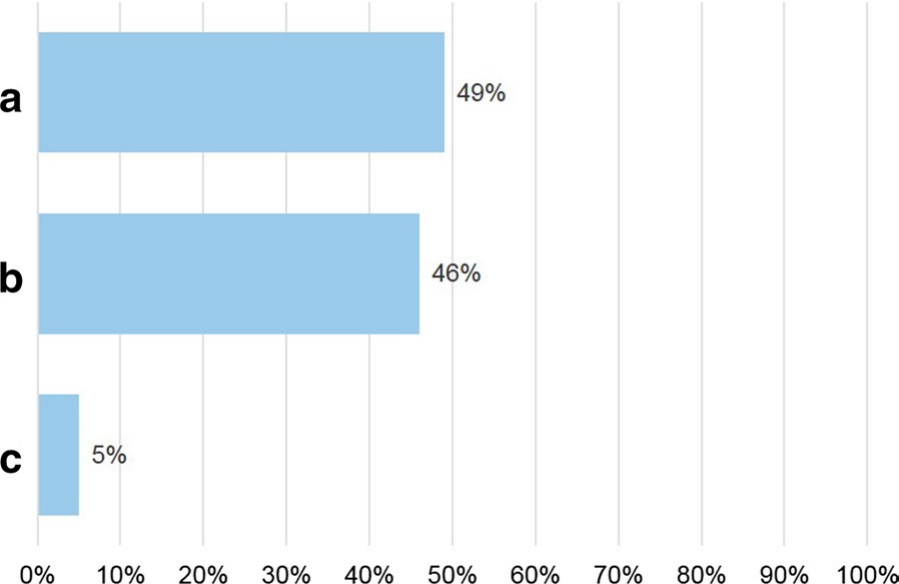
Answers to the question “According to your experience, the registered sublingual immunotherapy has a tolerability and safety profile”

**Fig. 5:**
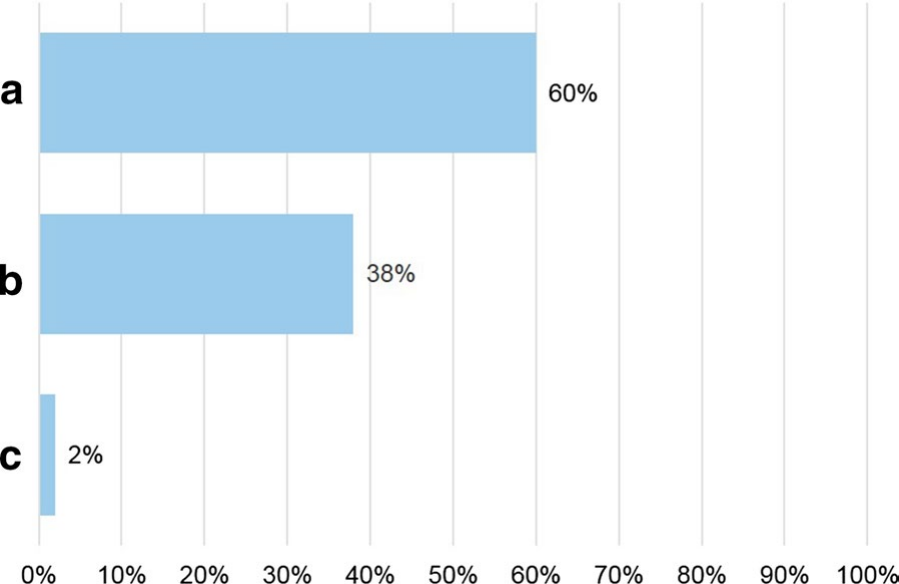
Answers to the question “In performing sublingual immunotherapy, is the build-up phase important?”

**Fig. 6:**
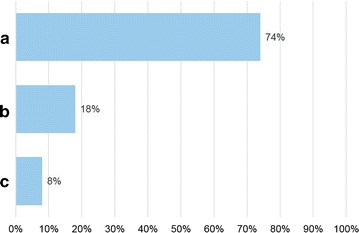
Answers to the question “After starting registered sublingual immunotherapy, do you check the patient after some weeks/months?”

## Discussion

The registration of grass pollen tablets as pharmaceutical quality products for grass pollen-induced rhinitis was a breakthrough in the history of AIT [7]. In fact, the commonly used AIT products were defined as Named Patient Products (NPP), because they were individual preparations that were not considered by the regulatory agencies as registered products [8]. The large placebo-controlled trials with grass pollen tablets performed to obtain the authorization by EMA as registered products clearly demonstrated the efficacy and safety of SLIT and paved the way to the modern AIT [9, 10]. In particular, the registration of the 5-grass-pollen tablets, as well as the data obtained from post marketing studies dealing with pharmacoeconomic impact [11] and patients’ phenotyping [12] has led to the approval for full reimbursement by AIFA as a treatment for seasonal allergic rhinitis. Soon after, the one-grass (*Phleum pratense*) pollen tablet was also approved. In this survey we evaluated the opinions of the Italian allergy specialists about the reimbursed grass-pollen tablets, including the differences between the 5 grass-pollen and the 1-grass pollen tablets. The number of allergy specialists participating to the survey was not large, but the decision to fill in the questionnaire was spontaneous and no repeated invitation was sent. The overall opinion on the grass-pollen tablets identified as key factors the concept of AIT products as drugs (indicated by 51% of the allergists) and the utility of patient’s monitoring after starting the treatment (indicated by 74% of the allergists). The other four issues allowed to highlight the perceived differences between the two products. Concerning the question assessing the value of an allergen content mirroring the natural exposure of patients to grass pollen, 92% of the allergists attributed to such factor a pivotal importance. Indeed, the superior adequacy of SLIT by a mix of five grasses over only *P. pratense* in Italian patients was indicated by both botanical and immunological studies. From the botanical point of view, phenologic analyses were performed on a number of grasses, by sampling every 10 days, starting in April, in 50 stations distributed across Italy. The flowering phase was assessed using a stereomicroscopy-based method for the detection of spreading stamens, and the data were compared to those from the official pollen calendar. Relevant differences were found between grass pollen count and effective flowering of the grass species as assessed by phenology. In fact, only some species contributed to the pollen peak, while important *Pooideae*, such as *P. pratense*, were not present during the pollen peak in northern and central Italy [13]. Immunological data supported the botanical observation, based on the results obtained with RAST-inhibition using sera of grass allergic patients from central Italy, that showed a significantly higher binding by the 5-grass extract compared with the *P. pratense* extract when grass pollens other than *P. pratense* were tested [14]. The questions concerning the pre-coseasonal schedule as the most patient’s oriented, the profile of tolerability and safety and the importance of the build-up phase pertain to the same issue. In fact, a pre-coseasonal protocol, as indicated for the 5-grass pollen tablets, is defined as optimal for pollen allergy [15], while the protocol indicated for *P. pratense* tablet is perennial. In addition, the build-up phase and the safety are apparently related, as shown by the fact that the majority of severe adverse reactions occurred with the administration of the first dose of the *P. pratense* tablet, that has no build-up phase [16, 17]. On the other hand, in the position paper endorsed by the World Health Organization allergen immunotherapy is defined as “the practice of administering gradually increasing quantities of an allergen extract to an allergic subject to ameliorate the symptoms associated with subsequent exposure to the causative allergen” [18]. Thus, an AIT schedule with no build-up phase is not adherent to such definition.

In conclusion, the opinions of the Italian allergy specialists about the reimbursed 5-grass-pollen tablets are very positive and make likely an appropriate prescription of SLIT for grass-pollen induced rhinitis in the next years. Expanding this kind of survey to other specialists and including in the questionnaires also a comparison between AIT registered tablets and the common NPP is likely to further improve our knowledge on physicians opinion on AIT.
